# Structural Relationship and Binding Mechanisms of Five Flavonoids with Bovine Serum Albumin 

**DOI:** 10.3390/molecules15129092

**Published:** 2010-12-09

**Authors:** E-Hu Liu, Lian-Wen Qi, Ping Li

**Affiliations:** 1 Key Laboratory of Modern Chinese Medicines (China Pharmaceutical University), Ministry of Education, Nanjing 210009, China; 2 Department of Pharmacognosy, College of Pharmacy, 3rd Military Medical University, Chongqing, China

**Keywords:** flavonoids, bovine serum albumin, fluorescence quenching, binding mechanism

## Abstract

Flavonoids are structurally diverse and the most ubiquitous groups of dietary polyphenols distributed in various fruits and vegetables. In this study, the interaction between five flavonoids, namely formononetin-7-*O*-β-D-glucoside, calycosin- 7-*O*-β-D-glucoside, calycosin, rutin, and quercetin, and bovine serum albumin (BSA) was investigated by fluorescence and UV-vis absorbance spectroscopy. In the discussion, it was proved that the fluorescence quenching of BSA by flavonoids was a result of the formation of a flavonoid–BSA complex. Fluorescence quenching constants were determined using the Stern-Volmer and Lineweaver-Burk equations to provide a measure of the binding affinity between the flavonoids and BSA. The binding constants ranked in the order quercetin > rutin > calycosin > calycosin-7-*O*-β-D-glucoside ≈ formononetin-7-*O*-β-D-glucoside. The results of thermodynamic parameters Δ*G*, Δ*H*, and Δ*S* at different temperatures indicated that the hydrophobic interaction played a major role in flavonoid–BSA association. The distance *r* between BSA and acceptor flavonoids was also obtained according to Förster’s theory of non-radiative energy transfer.

## 1. Introduction

Flavonoids are a large group of phytochemicals with a chromane-type skeleton and a phenyl substituent in the C_2_ or C_3_ position. They have attracted considerable attention, specifically because flavonoids are of biological and physiological importance. Plant flavonoids have been shown to modify eicosanoid biosynthesis, protect low-density lipoproteins from oxidation, prevent platelet aggregation, and promote relaxation of cardiovascular smooth muscle. Flavonoids have also been shown to have antiviral, cytotoxic, anti-ulcerogenic, and carcinostatic activities [1,2].

Proteins are frequently considered major ‘targets’ for therapeutically active flavonoids [3]. Serum albumins are the most abundant proteins in the circulatory system and have many physiological functions. The research on drug–protein interactions is an active field of interest because of the prospective of unraveling of drug action mechanisms and the possibility of designing novel medicines. Plasma protein binding is an important factor to understand the pharmacokinetics and pharmacodynamic properties of drug candidates, because it strongly influences drug distribution and determines the free fraction.

**Figure 1:**
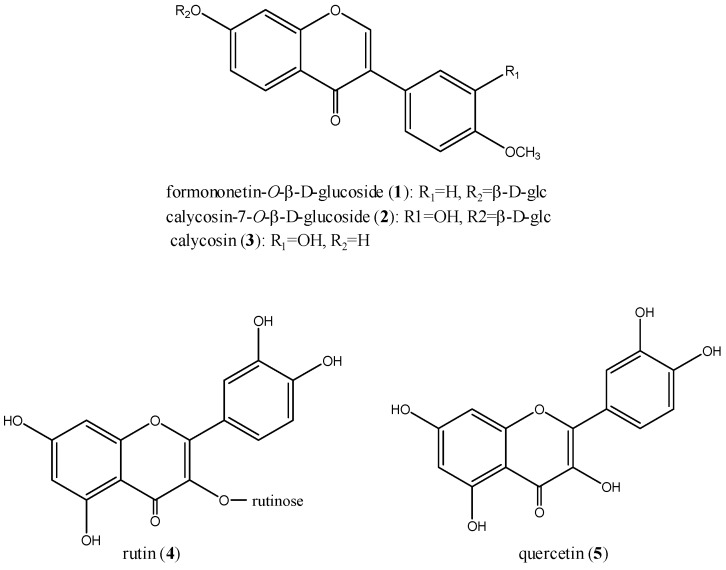
Chemical structures of the five flavonoids.

Spectral methods are powerful tools for the study of drug binding with proteins since they allow nonintrusive measurements of substances in low concentration under physiological conditions. There have been several studies on fluorescence quenching of proteins induced by flavonoids and other polyphenols [4,5,6,7,8,9,10,11,12,13,14,15,16,17]. The mode of interaction and detailed binding mechanism of the flavonoid compounds with their target proteins at molecule level are gradually being understood. However, there have been few investigations on the influence of hydroxylation and glycosylation of flavones and isoflavones and their binding characteristic with proteins. Furthermore, few data are available on the differences in binding of flavonoids and isoflavonoids.

In this work, the binding properties of five flavonoids, *i.e.,* formononetin-7-*O*-β-D-glucoside, calycosin-7-*O*-β-D-glucoside, calycosin, rutin, and quercetin, (Figure 1) to BSA were investigated by fluorescence and UV–vis absorption spectroscopy. The thermodynamic properties and the binding parameters of flavonoids with BSA such as quenching rate constants, binding modes, binding constants, binding sites, and intermolecular distances have been described. The present work is expected to provide a quantitative understanding of the effect of flavonoids on the structure of BSA. It is also interesting to study the structural-binding affinity relationships of flavonoids and isoflavonoids with serum albumins.

## 2. Results and Discussion

### 2.1. Fluorescence quenching mechanism

Quenching can occur by different mechanisms which are usually classified as dynamic quenching and static quenching, which can be distinguished by their differing dependence on temperature and viscosity, or preferably by lifetime measurements [18]. Higher temperatures result in faster diffusion and hence larger extent of collisional quenching. Higher temperatures will also result in the dissociation of weakly bound complexes, and hence lead to less static quenching.

**Figure 2:**
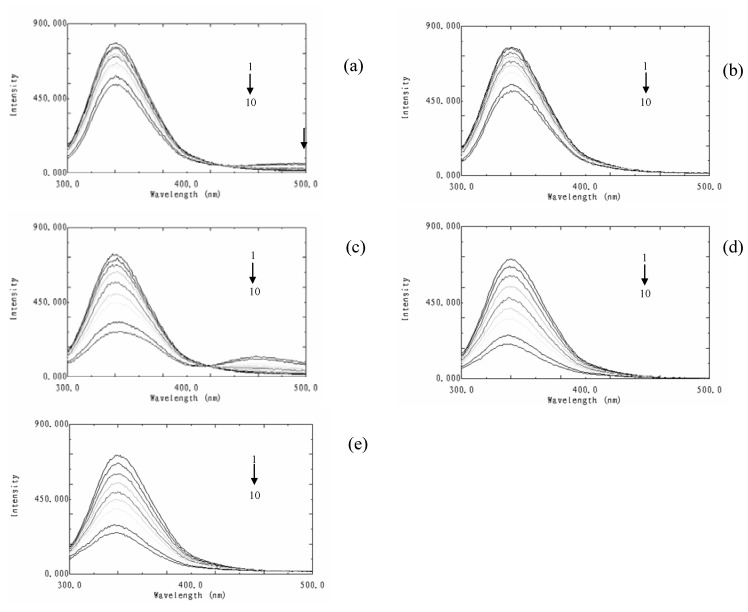
Fluorescence emission spectra of BSA in the presence of various concentrations of flavonoids. (a): formononetin-7-*O*-β-D-glucoside; (b): calycosin-7-*O*-β-D-glucoside; (c): calyosin; (d): rutin, (e): quercetin. T = 298 K, λex = 280 nm; c(BSA) = 2.0 ×10^-6^ mol·L^−1^; c(flavonoid), 1-10: 0, 0.67, 2.00, 4.00, 6.67, 10.00, 14.00, 18.67, 24.00, 30.00 μM.

As shown in Figure 2, BSA showed strong fluorescence emission while the flavonoids displayed almost no intrinsic fluorescence under the experimental conditions used. The fluorescence intensities of BSA decreased with increasing flavonoid concentrations, accompanied by a weak blue shift of the maximum emission wavelengths in the fluorescence spectra. These data showed that flavonoids could interact with BSA and quench their intrinsic fluorescence. The blue shift signified that the binding of flavonoids was associated with some change in the immediate environment of the tryptophan residues and the fact that the flavonoids were situated at close proximity to the tryptophan residue for quenching to occur [14].

To confirm the quenching mechanism, we analyzed the fluorescence data at different temperatures with the well-known Stern-Volmer (1) and Lineweaver-Burk (2) equations. The two equations are commonly used in describing dynamic quenching and static quenching, respectively.

*F*_0_ / *F* = 1 + *K*_q_*τ*_0_[Q] = 1 + *K*_SV_[Q] (1)

where *F*_0_ and *F* are the fluorescence intensities in the absence and presence of quencher, respectively; *K*_q_ is the bimolecular quenching constant; *τ*_0_ is the lifetime of the fluorophore in the absence of quencher and its value is 10^-8^ s; [Q] is the concentration of quencher; and *K*sv is the Stern–Volmer quenching constant which can be obtained by the slope (see Table 1). These results show that the Stern-Volmer quenching constant *K*_SV_ is inversely correlated with temperature, which indicates that the probable quenching mechanism of the flavonoid–BSA binding reaction is initiated by compound formation rather than by dynamic collision. Moreover, the values of *K*_q_ in Table 1 fell in the range of 3.60 × 10^12^ to 2.18 × 10^13^ mol·L^-1^ for all reactions of flavonoid–BSA and were far larger than 2.0 × 10^10^ mol·L^-1^, the maximum diffusion collision quenching rate constant of various quenchers with the biopolymer [19]. Consequently, the probable quenching mechanism of fluorescence of the flavonoids–BSA binding reactions should follow a static quenching process rather than a dynamic one.

**Table 1:** Stern-Volmer quenching constants for the interactions of flavonoids with BSA at different temperatures.

Compound	*T*/(K)	*K*_SV _/ (L·mol^ - 1^)
formononetin-7-*O*-β-D-glucoside	298	1.53 × 10^4^ (r^2^ = 0.9883)
	310	1.4 × 10^4^ (r^2^ = 0.9947)
calycosin-7-*O*-β-D-glucoside	298	1.66 × 10^4^ (r^2^ = 0.9944)
	310	1.4 × 10^4^ (r^2^ = 0.9917)
calyosin	298	5.51 × 10^4^ (r^2^ = 0.9955)
	310	4.88 × 10^4^ (r^2^ = 0.9954)
rutin	298	7.88 × 10^4^ (r^2^ = 0.9909)
	310	7.63 × 10^4^ (r^2^ = 0.9939)
quercetin	298	6.08 × 10^4^ (r^2^ = 0.9954)
	310	5.95 × 10^4^ (r^2^ = 0.9945)

Therefore, the quenching data were analyzed according to the modified Lineweaver-Burk equation:

(F_0_ - F)^-1^ = F_0_^-1^+KLB^-1^·F_0_^-1^·[Q] ^-1^ (2)

where *F*_0_ and *F* are the fluorescence intensities in the absence and in the presence of quencher at concentration [*Q*]; and *K*_LB_ is the effective quenching constant for the accessible fluorophores.

**Figure 3:**
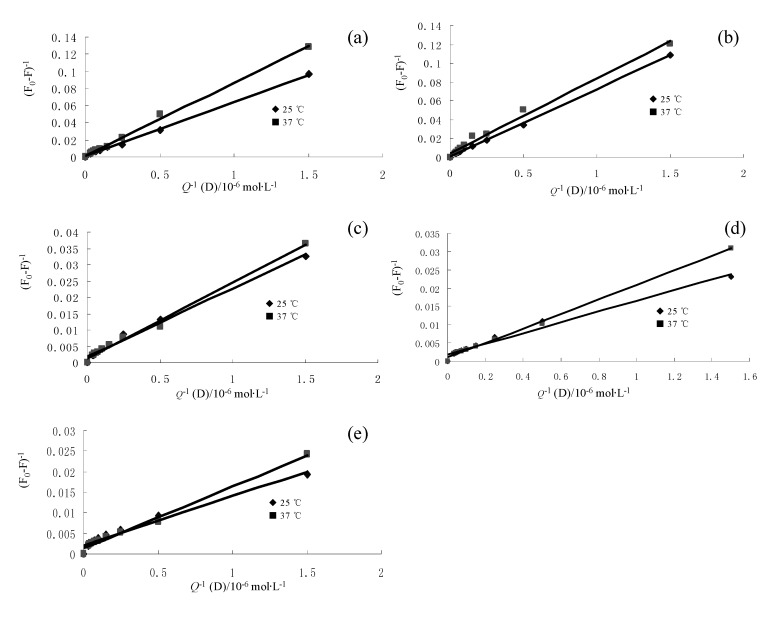
Lineweaver-burk plots of flavonoid–BSA systems (a) formononetin-7-*O*-β-D-glucoside; (b) calycosin-7-*O*-β-D-glucoside; (c) calycosin; (d) rutin, (e) quercetin.

The corresponding results at different temperatures are shown in Table 2. The decreasing trend of *K*_LB_ with increasing temperature is in accordance with *K*_SV_’s dependence on temperature (Figure 3). It shows that the binding constant between these flavonoids and BSA is moderate and the effect of temperature is not significant. Thus, these flavonoids can be stored and carried by this protein under physiological conditions.

**Table 2:** Lineweaver-burk quenching constants for the interactions of flavonoids with BSA at different temperatures.

Compound	*T*/(K)	*K*_LB _/ (L·mol ^- 1^)
formononetin-7-*O*-β-D-glucoside	298	0. 204 × 10^5^ (r^2^=0.9976)
	310	0.149 × 10^5^ (r^2^=0.9969)
calycosin-7-*O*-β-D-glucoside	298	0.184 × 10^5^ (r^2^=0.9990)
	310	0.178 × 10^5^ (r^2^=0. 9898)
calyosin	298	0.640 × 10^5^ (r^2^=0.9896)
	310	0.544 × 10^5^ (r^2^=0.9933)
rutin	298	0.973 × 10^5^ (r^2^=0.9802)
	310	0.707 × 10^5^ (r^2^=0.9974)
quercetin	298	1.170 × 10^5^ (r^2^=0.9739)
	310	1.134 × 10^5^ (r^2^=0.9909)

As can be seen from Table 2, the quenching constants *K*_LB_ ranked in the following order: quercetin > rutin > calycosin > calycosin-7-*O*-β-D-glucoside ≈ formononetin- 7-*O*-β-D-glucoside. The fluorescence quenching of BSA by flavonoids (quercetin and rutin) is stronger than that of isoflavonoids (calycosin, calycosin-7-*O*-β-D-glucoside, formononetin-7-*O*-β-D-glucoside). For rutin and its aglycone quercetin, the steric hindrance may take place after glycosylation at the 3- position, which weakened the binding affinity. Similarly, after glycosylation at the 7- position, the fluorescence quenching of BSA by calycosin, the isoflavone aglycone, is stronger than that of two isoflavonoid glycosides, calycosin-7-*O*-β-D-glucoside and formononetin-7-*O*-β-D-glucoside. Alternatively, the increased molecular weight of isoflavone glycosylation and polarity may reduce the capacity to penetrate into the tryptophan rich hydrophobic regions of BSA.

### 2.2. Determination of the binding mode between flavonoids and BSA

The interaction forces between drugs and biomolecules may include electrostatic interactions, multiple hydrogen bonds, van der Waals interactions, hydrophobic and steric contacts within the antibody-binding site, *etc* [20]. The thermodynamic parameters of binding reaction are considered as evidence for confirming the binding mode. To elucidate the interaction between these five flavonoids and BSA, the thermodynamic parameters were calculated from the van’t Hoff plots based on the temperature dependence of the binding constant for flavonoids -HSA binding.

If the enthalpy change (Δ*H*) does not vary significantly in the temperature range studied, both the enthalpy change (Δ*H*) and entropy change (Δ*S*) can be evaluated from the van’t Hoff equation:

ln*K* = Δ*H*/*RT*-Δ*S*/*R* (3)

where *K* is analogous to the associative binding constants at the corresponding temperature (298 and 310 K) and *R* is the gas constant. From the linear relationship between ln *K* and the reciprocal absolute temperature the value of Δ*H* and Δ*S* can be obtained. The free energy change (Δ*G*) is then estimated from the following equation:

Δ*G* = Δ*H* - *T*Δ*S* (4)

The values of Δ*H* and Δ*S* obtained for the binding site from the slopes and ordinates at the origin of the fitted lines are summarized in Table 3. The negative values of free energy (Δ*G*) indicate that all binding processes are spontaneous. For typical hydrophobic interactions, both Δ*H* and Δ*S* are positive, while negative enthalpy and entropy changes arise from van der Waals force and hydrogen bonding formation in low dielectric media. Based on studies of water structure, a positive entropy change is generally considered as evidence for hydrophobic interactions. In addition, a specific electrostatic interaction between ionic species in an aqueous solution is characterized by a positive Δ*S* value and negative Δ*H* value [21]. Accordingly, it was not possible to account for the thermodynamic parameters of flavonoid–BSA compounds on the basis of a single intermolecular force model. As shown in table 3, the main sources of Δ*G* values are derived from a large contribution of Δ*S* term with little contribution from the Δ*H* factor. All these results meant that the hydrophobic interaction might play a key role in the interaction between these five flavonoids and BSA, but the electrostatic interaction could not be excluded.

**Table 3:** Thermodynamic parameters of flavonoids –BSA binding systemat different temperatures.

Compound	*T*/(K)	Δ*H*/(kJ·mol ^-1^)	Δ*G*/ (kJ·mol ^-1^)	Δ*S*/(J·mol ^-1^·K^-1^)
formononetin-7- *O*-β-D-glucoside	298	-20.01	-24.59	15.37
	310		-24.77	15.99
calycosin-7- *O*-β-D-glucoside	298	-2.23	-24.33	74.16
	310		-25.22	77.15
calycosin	298	-10.41	-27.42	57.06
	310		-28.10	59.36
rutin	298	-20.48	-28.46	26.78
	310		-28.78	27.85
quercetin	298	-2.00	-28. 91	90.32
	298		-30. 00	93.96

### 2.3. Binding constants and binding sites

For the static quenching, if there are independent binding sites to a set of equivalent sites on a macromolecule, the number of sites *n* can be obtained from the following equation [22,23]:

 lg(*F*_0_-*F*)/*F* = lg*K_A_*+*n*lg[Q] (5) )

For the flavonoid and BSA system, the values of *K_A_* and *n* of these flavonoids at different temperatures are shown in Table 4, respectively. According to Equation (5), we can calculate that the number of binding sites between BSA and flavonoid is approximately equal to 1, which indicates that there is only one flavonoid molecule binding to one BSA molecule. All correlation coefficients are larger than 0.99, indicating that the interactions between flavonoids and BSA are consistent with the site-binding model. As shown in Table 4, binding constants of flavonoid–BSA decreased in the following order: quercetin > rutin > calycosin > calycosin-7-*O*-β-D-glucoside ≈ formononetin-7-*O*- β-D-glucoside.

**Table 4:** Binding parameters and relative thermodynamic parameters of the systems of flavonoid–BSA at different temperatures.

Compound	*T*/(K)	lg( *F*_0_-*F) /F* - lg[Q ]	*K*_A_/L·mol^-1^	n
formononetin-7- *O*-β-D-glucoside	298	y = 0.902x - 1.6545 (R^2^ = 0.9902)	0.22156 × 10^5^	0.902
	310	y = 0.9964x - 1.8236 (R^2^ = 0.9944)	0.15011 × 10^5^	0.9964
calycosin-7- *O*-β-D-glucoside	298	y = 0.96x - 1.7246 (R^2^ = 0.9969)	0.18854 × 10^5^	0.960
	310	y = 0.9376x - 1.8157 (R^2^ = 0.9863)	0.15286 × 10^5^	0.9376
calycosin	298	y = 0.9675x - 1.2526 (R^2^ = 0.9931)	0.55898 × 10^5^	0.9675
	310	y = 0.9474x - 1.2613 (R^2^ = 0.9929)	0.54780 × 10^5^	0.9474
rutin	298	y = 0.9518x - 1.0764 (R^2^ = 0.9936)	0.83869 × 10^5^	0.9518
	310	y = 1.0047x - 1.1281 (R^2^ = 0.9981)	0.74456 × 10^5^	1.0047
quercetin	298	y = 0.8238x - 0.9928 (R^2^ = 0.9950)	1.01671 × 10^5^	0.8238
	310	y = 0.8548x - 1.0053 (R^2^= 0.9916)	0.98787 × 10^5^	0.8548

Equations (1), (2) and (5) have been used by many researchers to investigate the interaction and binding mechanisms of fluorophore and quencher. Equations (1) and (2) are commonly used in describing dynamic quenching and static quenching, respectively and they are based on different models. Equation (1) is valid for dynamic quenching, while Equation (2) is valid if fluorophore and quencher form a 1:1 complex and if the fluorophore does not fluoresce at all once the complex is formed. For static quenching, there is only one quencher molecule binding to one fluorophore molecule (*n* = 1) and they form a 1:1 complex, the quenching constant for fluorophores is also the binding constant of the reaction. For *n* ≈ 1, Equation (5) can be rewritten to the following Equation (2).

### 2.4. Energy transfer from BSA to flavonoids

Fluorescence resonance energy transfer (FRET) is a spectroscopic method that can monitor the proximity and relative angular orientation of fluorophores. The donor and acceptor fluorophores can be entirely separated or attached to the same macromolecule. According to Förster’s non-radiative energy transfer theory, the energy transfer will happen under the following conditions: (a) the donor can produce fluorescence light; (b) fluorescence emission spectrum of the donor and absorbance spectrum of the acceptor have partial overlap; (c) the distance between the donor and the acceptor is less than approximately 8 nm (energy transfer efficiency varies inversely with the sixth power of the donor-acceptor distance) [24]. Using FRET, the distance *r* between flavonoids and BSA could be calculated by the following equation

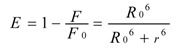
(6)
where *E* denotes the efficiency of transfer between the donor and the acceptor, *r* is the average distance between donor and acceptor, and *R*_0_ is the critical distance when the efficiency of transfer is 50%:.

**Figure 4:**
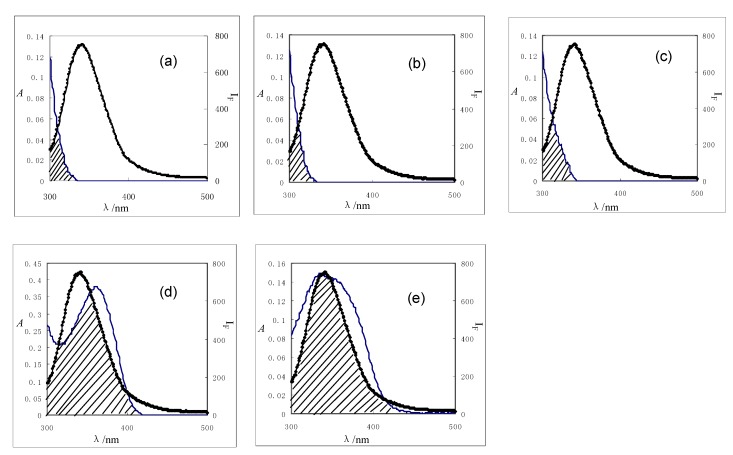
Overlaps of the fluorescence spectra (2) of BSA with the absorption spectra (1) of five flavonoids (a) formononetin-7-*O*-β-D-glucoside; (b) calycosin-7-*O*-β-D-glucoside; (c) calyosin; (d) rutin, (e) quercetin; c (BSA) = c (flavonoid) = 2.0 μM (T = 298 K).

*R*^6^_0_ = 8.8 × 10^-25^ (*K*^2^·*Φ*·*n*^-4^·*J*) (7)

where *K*^2^ is the orientation factor related to the geometry of the donor and acceptor dipoles and *K*^2^ = 2/3 for random orientation in solution; *n* is the average refractive index of the medium in the wavelength range where spectral overlap is significant; *φ* is the fluorescence quantum yield of the donor; *J* is the overlap integral between the emission spectrum of the donor and the absorption spectrum of the acceptor (Figure 4), which can be calculated by the following equation:

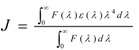
(8)
where *F*(*λ*) is the corrected fluorescence intensity of the donor in the wavelength range from *λ* to *λ* + Δ*λ* and *ε*(*λ*) is the extinction coefficient of the acceptor at *λ*.

Based on these data, the *J* values calculated by equation 8 were 4.67 × 10^-15^, 3.36 × 10^-15^, 3.49 × 10^-15^, 1.97 × 10^-13^, and 8.75 × 10^-14^ cm^3^·L·mol^-1^ for formononetin-7-*O*-β-D-glucoside, calycosin-7-*O*-β-D-glucoside, calycosin, rutin, and quercetin, respectively. The distances between flavonoids and BSA were obtained from equation 6. The values of *r* were 4.12 for formononetin-7-*O*-β-D-glucoside, 3.85 for calycosin-7-*O*-β-D-glucoside, 3.01 for calycosin, 5.72 for rutin, and 4.75 nm for quercetin. The average distances between a donor fluorophore and acceptor fluorophore are on the 2-to-8 nm scale, indicating that the energy transfer from BSA to flavonoids occurred with high probability [25].

## 3. Experimental

### 3.1. Apparatus

UV spectra were recorded on a UV-2501PC spectrophotometer (Shimadzu, Japan) equipped with 1.0 cm quartz cells. All fluorescence spectra were recorded on a RF-5301PC spectrofluorophotometer (Shimadzu, Japan) equipped with 1.0 cm quartz cells and a thermostat bath. Both the excitation slit and emission slit were set to 3 nm. The emission spectra were recorded between 300 and 500 nm and the excitation wavelength was set at 280 nm. Appropriate blanks corresponding to the buffer were subtracted to correct for fluorescence background.

### 3.2. Reagents

BSA was purchased from Sigma Chemical Company (USA); formononetin-7-*O*-β-D-glucoside, calycosin-7-*O*-β-D-glucoside, calycosin, rutin, and quercetin were isolated by the authors from the dried roots of *Astragalus membranaceus* (Fisch.) Bge. var. *mongholicus* (Bge.) Hsiao. Their structures (Figure 1) were determined and unequivocally identified by mass spectrometry (MS) and nuclear magnetic resonance (NMR) spectroscopy. Their purities were over 97% by normalization of the peak areas detected by HPLC-UV.

Tris–HCl (0.05 M) buffer solution containing 0.1 M NaCl was used to keep the pH of the solution at 7.40. BSA stock solution was prepared with the Tris–HCl buffer solution and kept in the dark at 4 °C. The stock solutions (1.0 mM) of flavonoids were prepared in anhydrous methanol. All other reagents were of analytical grade and double-distilled water was used throughout the experiments.

### 3.3. Fluorescence and ultraviolet measurement

A solution (3.0 mL) containing appropriate concentrations of BSA was titrated in the Tris–HCl buffer solution (pH 7.40) by successive addition of flavonoids stock solution. Titrations were done manually by using trace syringes. The flavonoids were added from concentrated stock solutions so that the volume increment was negligible. All experiments were performed at two different temperatures (298, 310 K). The UV spectra were obtained by scanning the solution on the spectrophotometer with the wavelength range of 200–500 nm. The operations were carried out at room temperature.

## 4. Conclusions

In this work, the interaction of five flavonoids with BSA was studied by fluorescence and UV-visible absorption spectroscopy. We observed that most likely static quenching is involved in the quenching mechanism of fluorescence of BSA by these flavonoids. The binding reaction was spontaneous, and hydrophobic interaction played a major role in the reaction. Structural-binding affinity relationships showed that the fluorescence quenching of BSA by flavonoids was stronger than that of isoflavonoids, probably because glycosylation weakened the binding affinity. The obtained results provide useful knowledge on pharmacological applications of flavonoids and valuable information for designing of new drugs.
